# LncRNA ZEB1-AS1 regulates hepatocellular carcinoma progression by targeting miR-23c

**DOI:** 10.1186/s12957-021-02176-8

**Published:** 2021-04-17

**Authors:** Shuai Xue, Fengqin Lu, Chunhui Sun, Jingjing Zhao, Honghua Zhen, Xin Li

**Affiliations:** 1 Department of Health Care, Qilu Hospital (Qingdao), Cheeloo College of Medicine, Shandong University, Qingdao, 266035 China; 2 Department of Geratology, Jinan Zhangqiu District Hospital of TCM, Jinan, 250200 China; 3 Department of Hepatobiliary Surgery, The Third People’s Hospital of Qingdao, Qingdao, 266041 China; 4 Department of Surgery, Zhangqiu District People’s Hospital, Jinan, 250200 China; 5 Department of Respiratory, Zhangqiu District People’s Hospital, Jinan, 250200 China; 6 Health Care Office, Qingdao Hospital of Traditional Chinese Medicine, Qingdao Hiser Hospital, No.4 Renmin Road, Shibei District, Qingdao, 266033 China

**Keywords:** ZEB1-AS1, Hepatocellular carcinoma, miR-23c

## Abstract

**Background:**

It has been reported that long-chain non-coding RNA (lncRNA) zinc finger E-box binding homeobox 1 antisense 1 (ZEB1-AS1) is an oncogene in various cancers, including hepatocellular carcinoma (HCC). We investigated the role and mechanism of ZEB1-AS1 as a competitive endogenous RNA (ceRNA) combined with miR-23c in HCC cell proliferation and invasion.

**Methods:**

QRT-PCR was used to detect ZEB1-AS1 and miR-23c expressions in HCC tissues and cells. The dual luciferase reporter assay detected the targeted regulation of miR-23c and ZEB1-AS1. We also performed the correlation analysis of their expression in HCC tissues by the Spearman’s correlation analysis. 3-(4,5-dimethylthiazol-2-yl)-2,5-diphenyltetrazolium bromide (MTT) assay was used to detect the proliferation of hepatoma cells. Cell invasion was assessed by the Transwell assay.

**Results:**

QRT-PCR results indicated ZEB1-AS1 was upregulated and miR-23c was downregulated in HCC tissues and cell lines. ZEB1-AS1 knockdown hampered the proliferation and invasion of HCC cells. Dual luciferase reporter assay showed that miR-23c is a target of ZEB1-AS1, and ZEB1-AS1 was significantly negatively correlated with the miR-23c expression in HCC tissues. The results of MTT and Transwell assay showed that miR-23c inhibition restored the inhibitory effect of ZEB1-AS1 knockdown on HCC cells proliferation and invasion.

**Conclusions:**

As a ceRNA, lncRNA ZEB1-AS1 may play a vital role in inhibiting HCC progression through miR-23c, which will provide new clues and theoretical basis for the HCC diagnosis and treatment.

## Introduction

Hepatocellular carcinoma (HCC), with an increasing trend in its incidence, is a common primary liver cancer, ranking sixth among the most common tumors [1]. The occurrence of HCC is closely related to many factors (such as hepatitis B virus and hepatitis C virus infection, long-term alcohol abuse, liver cirrhosis, aflatoxin intake, and metabolic diseases), but its exact pathogenesis, especially the molecular mechanism, remains unclear [2]. The main treatments for HCC include liver resection, liver transplantation, transarterial chemoembolization, transarterial radioembolization, tumor ablation, and targeted drug therapy [3]. Although scientists have made significant advancements in the diagnosis and treatment of patients with HCC, its clinical prognosis is still not satisfactory [1]. The 5-year survival rate of HCC is only 18%, which is the second most deadly cancer after pancreatic cancer [2]. The main reason for the poor prognosis of HCC patients is the lack of effective early diagnosis. The occurrence and development of HCC is a complex process involving a series of key genetic and epigenetic changes [4]. Therefore, it is urgent to deeply study the pathogenesis of HCC and find tumor markers with high sensitivity and specificity in order to provide a more reliable basis for early diagnosis, effective treatment, and prognosis judgment of HCC.

LncRNA is a transcription of more than 200 nucleotides and has no protein coding ability [5]. Both lncRNAs and miRNAs are non-coding RNAs, and miRNAs play important role in tumorigenesis, immunity, and development [6]. LncRNA has always been considered as the “noise” of gene transcription without actual functions, but studies have found that lncRNA also has various functions, especially in tumors [7]. LncRNA can act as a competitive endogenous RNA (ceRNA) to competitively bind with miRNA to regulate miRNA expression and inhibit the regulatory effect of miRNA on target genes, among which there may be new mechanisms of tumorigenesis and development, and elucidating these mechanisms will provide new ideas for tumor diagnosis and treatment [8]. However, there are many types of lncRNA, a large number of lncRNAs have not been discovered, and many mechanisms related to lncRNA have not been elucidated.

More and more studies have shown that the dysregulation of lncRNA is involved in the development of tumors, including HCC [9]. The dysregulated lncRNA participates in the development of HCC through various biological processes (cell proliferation, apoptosis, metastasis and angiogenesis, etc.) [10]. For example, upregulation of CASC15 in HCC promoted tumor cell proliferation, migration and invasion, while hampered cell apoptosis [11]. CCAT2 facilitates to proliferation and metastasis in HCC [12]. SNHG3 overexpression is related to HCC malignant status and poor prognosis [13]. Thus, lncRNA could be used as a biomarker for HCC treatment and prognosis, providing a new way to evaluate the prognosis of HCC.

ZEB1-AS1 is a lncRNA which is widely associated with the development of various tumors. It was found to be involved in the pathophysiological processes of prostate cancer, colorectal cancer, bladder cancer, osteosarcoma, glioma, esophageal squamous cell carcinoma, B lymphocytic leukemia, and HCC [14]. ZEB1-AS1 expression was increased in HCC tissue, and it was also elevated in metastatic tumor tissue versus primary tumor tissue [15]. However, ZEB1-AS1 expression and its specific roles in HCC progression remain unclear. Thus, we explored the ZEB1-AS1 expression and its molecular regulatory mechanism involved in HCC, to provide new understandings for further research on the molecular mechanism and clinical molecular treatment of HCC.

## Material and methods

### Tissues samples

HCC and adjacent tissue (3–4 cm away from the cancer tissue) were collected from 32 patients treated in the oncology department of Qilu Hospital (Qingdao), Cheeloo College of Medicine, Shandong University, from June 2018 to September 2019. There were 20 males and 12 females. The age ranged from 25 to 68 years, with an average age of 55.8 ± 7.6 years. All patients provided written informed consent and the study was approved by the clinical research medical ethics committee of the hospital. The inclusion criteria are as follows: (1) no radiotherapy, chemotherapy, or immunotherapy before surgery; (2) primary liver cancer; and (3) no other malignant tumors or other systemic diseases. The exclusion criteria are as follows: (1) patients taking anti-tumor drugs or radiotherapy and other treatments and (2) patients with other malignant tumors.

### Cell culture

Human normal hepatocyte line MIHA and human HCC cell lines including Huh7, SK-HEP-1, SNU-387, JHH-7, and HCCLM3 were obtained from the Chinese Academy of Sciences Cell Bank (Shanghai, China). Cells were cultured in a cell incubator at 37 °C and 5% CO_2_ with DMEM (HyClone, USA) containing 10% fetal bovine serum (FBS, HyClone, USA) and 1% penicillin/streptomycin (Gibco, USA).

### Cell transfection

Small interfering RNAs of ZEB1-AS1 (si-ZEB1-AS1) and its control (si-NC), miR-23c inhibitor (inhibitor) and inhibitor control (anti-NC), and miR-23c mimic (mimic) and mimic control (miR-NC) were synthesized by GenePharma (Shanghai, China). Cells were seeded in 6-well plates with 1 × 10^5^/well. When the cell fusion reached 70–80%, they were replaced with a serum-free medium for culture. Lipofectamine^TM^ 3000 (Invitrogen, USA) was used for cell transfection experiment. After 48 h of transfection, the efficiency of transfection was evaluated through qRT-PCR.

### Cell proliferation assay

The cell proliferation of each group was detected by MTT assay. The cells were seeded in a 96-well plate with 1 × 10^4^ cells/well and cultured in a 37 °C, 5% CO_2_ constant temperature incubator. After being cultured for 1, 2, 3, and 4 d respectively, 10 μl of MTT solution (Sigma, USA) was added. After shaking and mixing, culture was continued for 4 h. One hundred fifty microliters of DMSO was added to terminate the reaction. The optical density (OD) at 490 nm was measured with a microplate reader.

### Transwell assay

The transfected cells were cultured for 24 h and digested with 0.25% EDTA trypsin to make cell suspension (4 × 10^5^ cells/ml). Two hundred microliters of serum-free cell suspension was added to the upper chamber of each pre-embedded Matrigel (Invitrogen, USA), and 600 μl of DMEM culture medium containing 10% FBS was filled in the lower chamber. After 24 h, the chamber was taken out and washed with PBS. The transmembrane cells were fixed with methanol for 20 min and then stained with crystal violet. The number of transmembrane cells was counted under an inverted microscope.

### Dual-luciferase reporter assay

The binding candidates of ZEB1-AS1 were predicted by bioinformatics analysis using starBase online (http://starbase.sysu.edu.cn/index.php). All parameters were default. The mutant type (mut: site-directed mutation) and wild type (wt) 3′-UTR fragment of ZEB1-AS1 containing the miR-23c seed sequence were cloned into the pGL3-basic vector (Invitrogen, USA) to form luciferase reporter plasmid (ZEB1-AS1-mut and ZEB1-AS1-wt) respectively. The constructed plasmid vector was co-transfected into cells with miR-NC or miR-23c mimic severally. After 48 h, the luciferase activity was detected by a dual luciferase reporter analysis system (Promega, USA).

### qRT-PCR

Trizol method was performed to extract total RNAs. The concentration and purity of the extracted RNA were detected by a NanoDrop spectrophotometer. The RNA was reverse transcribed into cDNA according to the system and conditions described in the reverse transcription kit (Takara, Japan). Real-time quantitative PCR experiments were performed on the Roche LightCycler480 (LC480) instrument using the SYBR Premix PCR kit (Takara, Japan) according to the instructions. The relative expression levels of RNAs were calculated by the 2^−△△Ct^ method. U6 was used as endogenous controls. The experiment was repeated independently three times.

### Statistical analysis

The data were expressed as mean ± SD. Statistical analysis was performed with GraphPad Prism 5 software. Comparison between the two groups was performed by t test. *P <* 0.05 indicated statistically significant.

## Results

### ZEB1-AS1 is upregulated in HCC

**Fig. 1:**
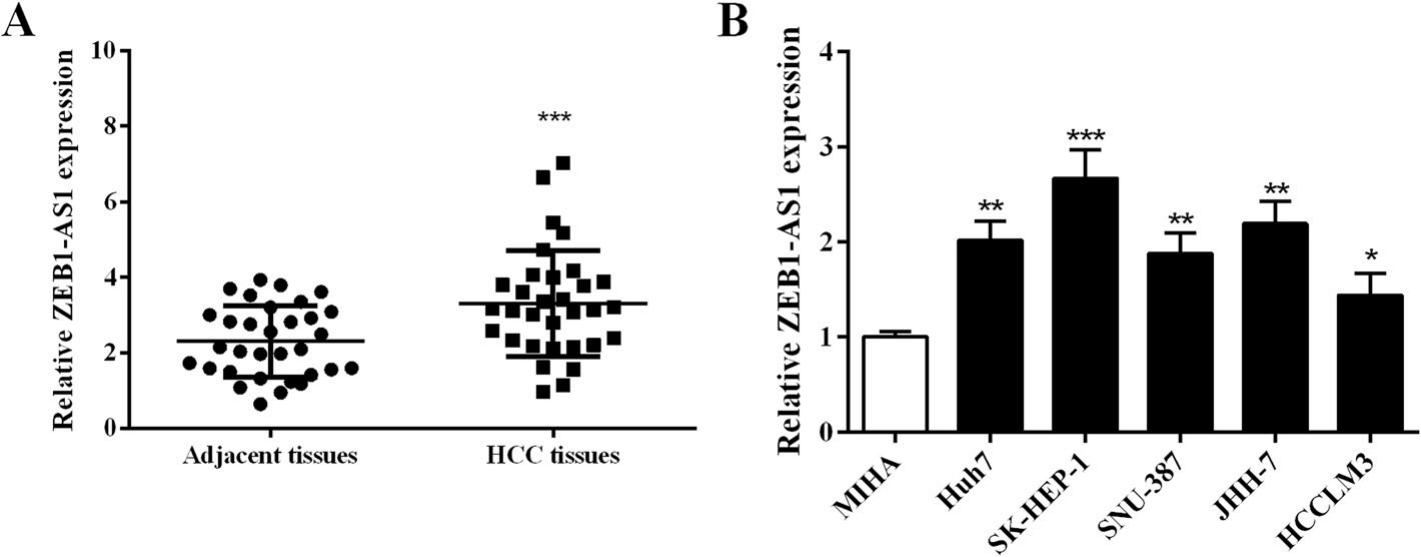
ZEB1-AS1 expression was analyzed in HCC tissues and cells by qRT-PCR. **a** ZEB1-AS1 expression was increased in HCC tissues. **b** ZEB1-AS1 expression was enhanced in human normal hepatocyte line MIHA and human HCC cell lines (Huh7, SK-HEP-1, SNU-387, JHH-7, and HCCLM3). **P < 0.01, ***P < 0.001

ZEB1-AS1 expression in the specimens of HCC and corresponding adjacent tissues was detected through qRT-PCR. The results revealed that ZEB1-AS1 was highly expressed in HCC versus adjacent tissues (Fig. 1a). Compared with the MIHA cell, the ZEB1-AS1 expression in HCC cells was notably increased (Fig. 1b). It was suggested that ZEB1-AS1 was highly expressed in HCC tissues and cells. ZEB1-AS1 showed a higher expression in SK-HEP-1 and JHH-7 cells, so they were selected for subsequent experiments.

### ZEB1-AS1 knockdown suppresses proliferation and invasion of HCC cell

**Fig. 2:**
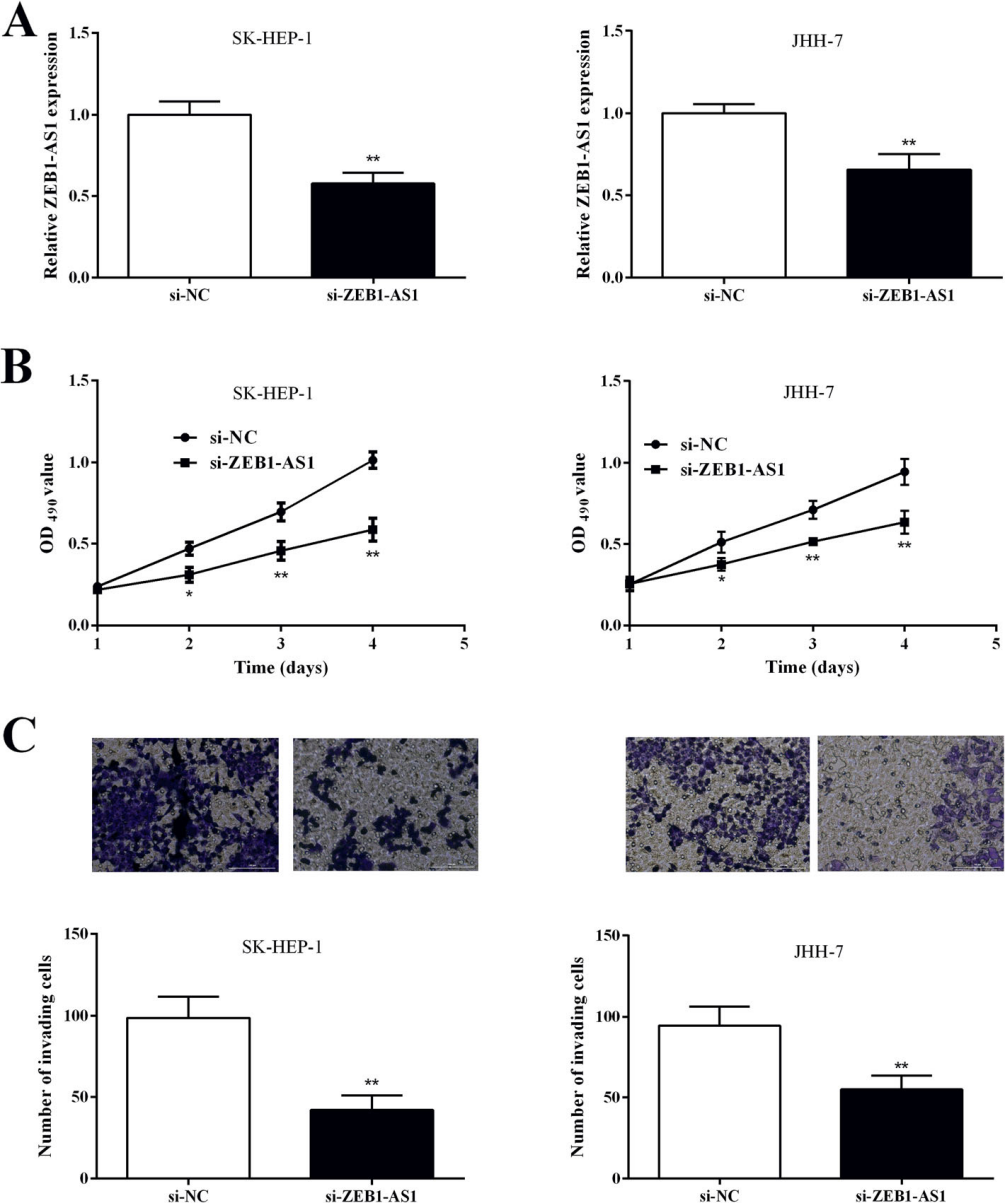
ZEB1-AS1 knockdown repressed HCC cell proliferation and invasion. **a** ZEB1-AS1 expression in SK-HEP-1 and JHH-7 cells transfected with si-ZEB1-AS1. **b** Cell proliferation was detected in SK-HEP-1 and JHH-7 cells treated by si-ZEB1-AS1 through MTT assay. **c** The number of invading cells were measured in SK-HEP-1 and JHH-7 cells transfected with si-ZEB1-AS1 by Transwell assay. *P < 0.05, **P < 0.01

To explore the role of ZEB1-AS1 in HCC cells, ZEB1-AS1 expression was interfered in HCC cells. We also observed the effect of ZEB1-AS1 inhibition on proliferation and invasion of HCC cells through MTT and Transwell experiments. After si-ZEB1-AS1 was transfected in SK-HEP-1 and JHH-7 cells respectively, qRT-PCR detection demonstrated that the ZEB1-AS1 expression in the si-ZEB1-AS1 group was significantly lower (0.577 ± 0.038, 0.657 ± 0.055) than that of the si-NC group (1.000 ± 0.047, 1.000 ± 0.032) (Fig. 2a). It showed that transfection of si-ZEB1-AS1 could effectively downregulate ZEB1-AS1 expression.

The cell proliferation in each group after transfection was detected by MTT experiment. As a result, the OD value of SK-HEP-1 and JHH-7 cells in the si-ZEB1-AS1 group (0.217 ± 0.025, 0.310 ± 0.046, 0.457 ± 0.057, 0.587 ± 0.070; 0.253 ± 0.045, 0.373 ± 0.038, 0.513 ± 0.025, 0.633 ± 0.071) was markedly reduced compared with si-NC group (0.237 ± 0.025, 0.470 ± 0.040, 0.697 ± 0.055, 1.013 ± 0.050; 0.250 ± 0.036, 0.510 ± 0.066, 0.710 ± 0.056, 0.943 ± 0.080) (Fig. 2b). This indicated that the ZEB1-AS1 knockdown inhibited the proliferation of SK-HEP-1 and JHH-7 cells. Similarly, the number of invasive cells in the si-ZEB1-AS1 group (42.000 ± 5.196, 55.000 ± 4.933) was obviously decreased versus the si-NC group (98.670 ± 7.513, 94.330 ± 6.936) (Fig. 2c). It indicated that the ZEB1-AS1 knockdown reduced the invasion ability of HCC cells.

### miR-23c directly binds to ZEB1-AS1

**Fig. 3:**
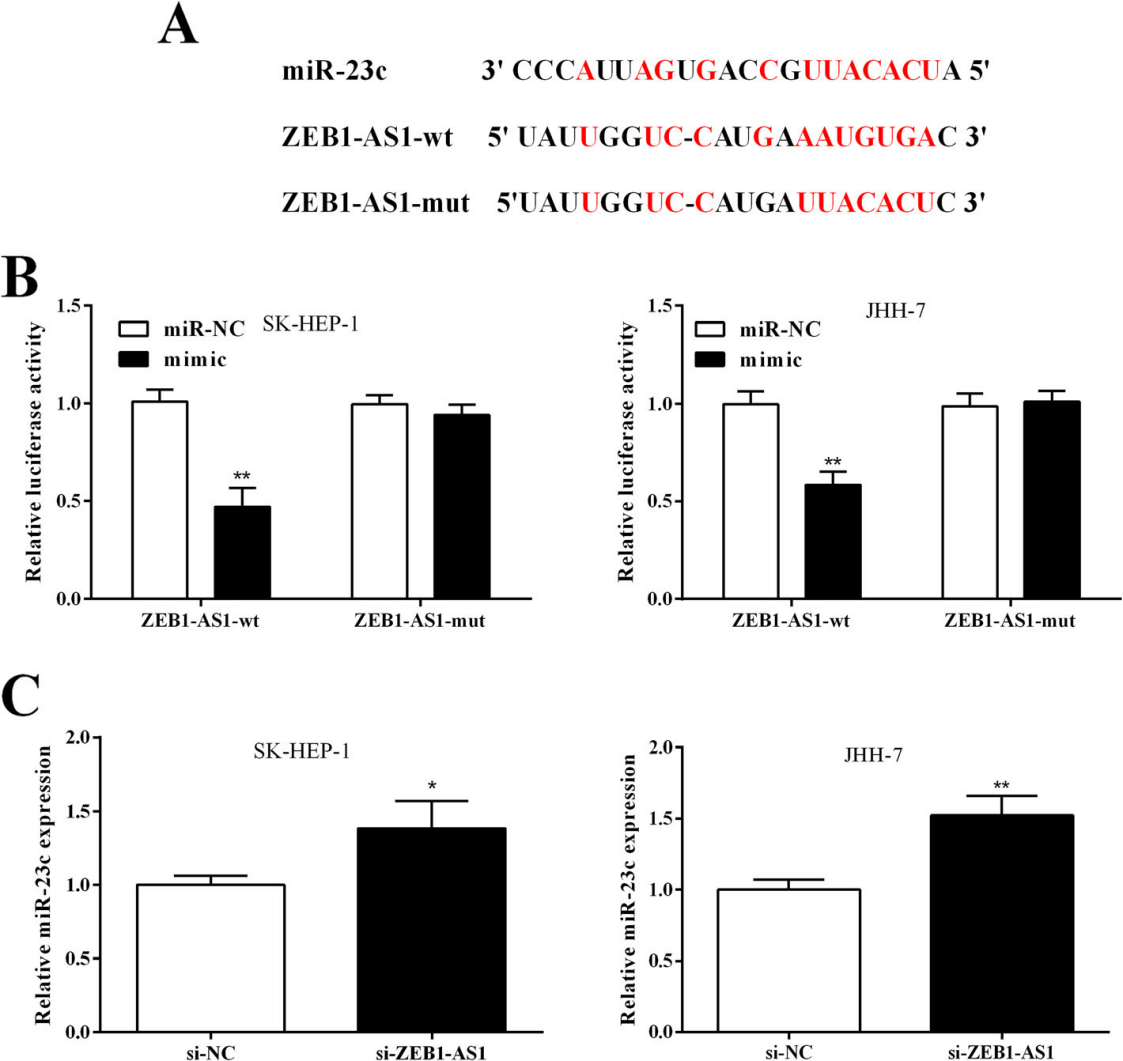
ZEB1-AS1 directly targeted miR-23c. **a** Potential binding sites of ZEB1-AS1 and miR-23c. **b** Luciferase activity was detected in SK-HEP-1 and JHH-7 cells. (C) miR-23c expression was elevated in SK-HEP-1 and JHH-7 cells transfected with si-ZEB1-AS1. **P < 0.01, ***P < 0.001

Bioinformatics analysis found that ZEB1-AS1 and miR-23c have binding sites, as shown in Fig. 3a. Double luciferase report experiments showed that luciferase activity was significantly decreased in ZEB1-AS1-wt and miR-23c mimic co-transfected SK-HEP-1 (0.470 ± 0.096) and JHH-7 (0.583 ± 0.068) cells versus ZEB1-AS1-wt and miR-23c miR-NC co-transfection (1.010 ± 0.060, 0.997 ± 0.067) (Fig. 3b). After ZEB1-AS1-mut and miR-23c mimic were co-transfected into SK-HEP-1 (0.940 ± 0.053) and JHH-7 (1.010 ± 0.056) cells respectively, luciferase activity did not change significantly versus ZEB1-AS1-wt and miR-23c miR-NC co-transfection (0.997 ± 0.047, 0.987 ± 0.064) (Fig. 3b). si-ZEB1-AS1 could significantly up-regulate miR-23c expression in SK-HEP-1 (1.383 ± 0.107) and JHH-7 (1.523 ± 0.078) cells compared with si-NC group (1.000 ± 0.035, 1.000 ± 0.042) (Fig. 3c). This indicated that there was a targeting relationship between ZEB1-AS1 and miR-23c.

### miR-23c is downregulated in HCC

**Fig. 4:**
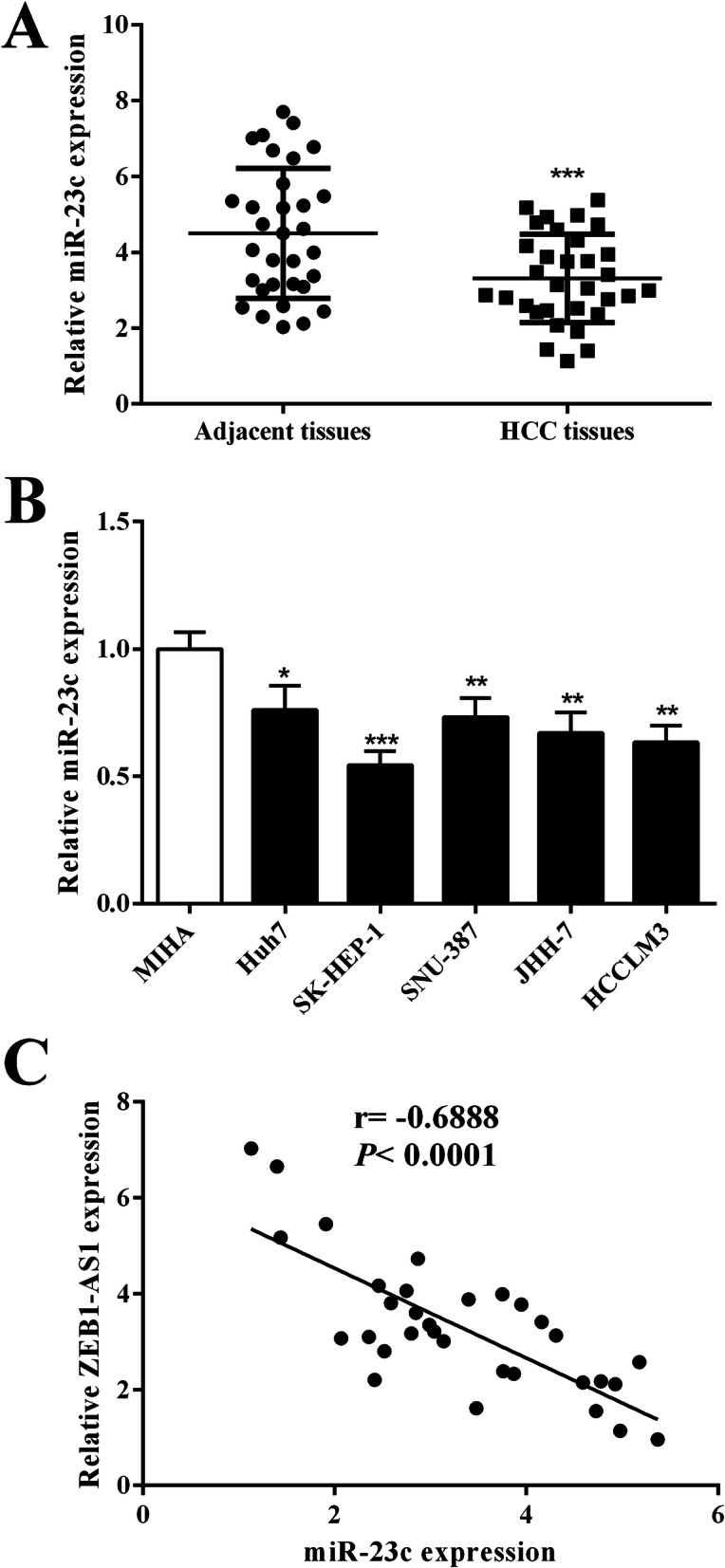
Expression of miR-23c was reduced in HCC tissues and cells. **a** Expression of miR-23cp was decreased in HCC tissue. **b** Expression of miR-23c was reduced in HCC cells. **c** The correlation analysis of ZEB1-AS1 and miR-23c expression in HCC tissues. **P < 0.01, ***P < 0.001

The miR-23c expression in HCC tissues was analyzed by qRT-PCR. The results showed that miR-23c expression in HCC tissues was markedly lower than that in adjacent tissues (Fig. 4a). Analysis of miR-23c expression in the HCC cell line indicated that miR-23c was downregulated significantly in the HCC cell line (Fig. 4b). In HCC tissue, correlation analysis of miR-23c expression and previous ZEB1-AS1 expression results were carried out using Spearman's correlation analysis. A significant negative correlation between the expression of ZEB1-AS1 and miR-23c in HCC tissues was observed (*r* = − 0.6888, *P <* 0.0001) (Fig. 4c).

### ZEB1-AS1 facilitates to proliferation and invasion of HCC cell through miR-23c

**Fig. 5:**
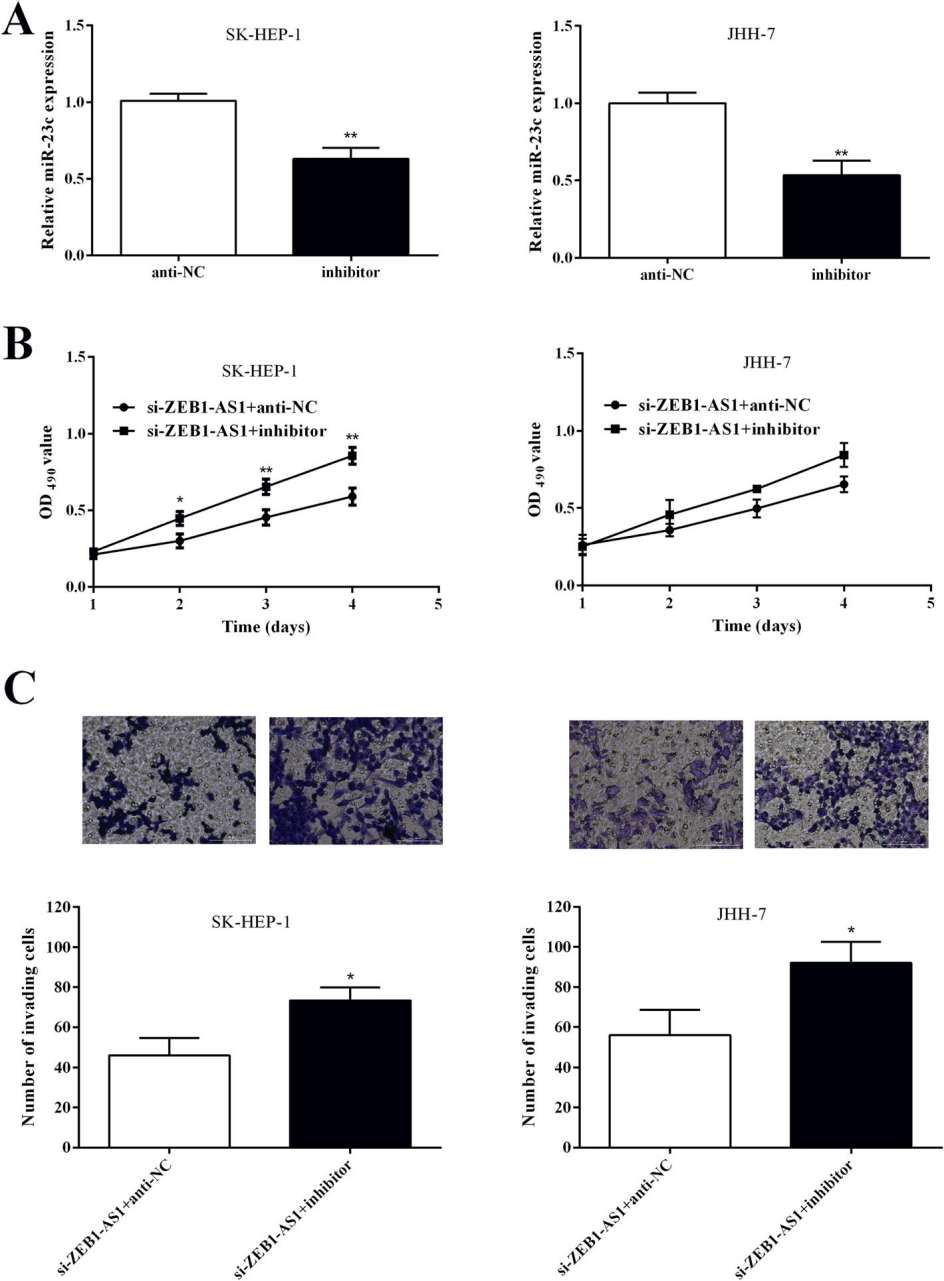
miR-23c inhibition reversed the suppressive effect of ZEB1-AS1 knockdown on HCC cell proliferation and invasion. (A) miR-23c expression in SK-HEP-1 and JHH-7 cells was decreased after the miR-23c inhibitor was transfected. (B) Cell proliferation was measured in SK-HEP-1 and JHH-7 cells transfected with si-ZEB1-AS1 and miR-23c inhibitor by MTT assay. (C) Invaded cells number of SK-HEP-1 and JHH-7 cells transfected with si-ZEB1-AS1 and miR-23c inhibitor was detected by Transwell assay. *P < 0.05, **P < 0.01

In order to verify whether ZEB1-AS1 affected the biological function of HCC cells through miR-23c targeted binding, the miR-23c inhibitor was transfected into SK-HEP-1 and JHH-7 cells, respectively. miR-23c expression was observably reduced in inhibitor group SK-HEP-1 (0.630 ± 0.042) and JHH-7 (0.533 ± 0.055) cells versus anti-NC group (1.010 ± 0.026, 1.000 ± 0.040) (Fig. 5a). We found that si-ZEB1-AS1 and miR-23c inhibitor co-transfection alleviated the inhibitory effect of si-ZEB1-AS1 on cell proliferation and invasion (Fig. 5b, c). It proved that ZEB1-AS1 played a role in HCC progression through miR-23c.

## Discussion

HCC, which is characterized by rapid progression, a high degree of malignancy, poor prognosis, and high mortality, is the most common pathological type of hepatic malignancy [16]. Increasing evidence shows that the occurrence of HCC is a multi-step process involving complex interactions between genetics, epigenetics, and transcriptomics [17]. The poor prognosis of HCC is related to various risk factors, such as HBV and HCV caused by chronic infection, a liver disease caused by alcohol, liver cirrhosis, and diabetes [18, 19]. There are also risks caused by endocrine factors, obesity, nutritional factors, environmental factors, and certain genetic diseases [20]. The complexity of HCC pathology makes it difficult to identify the main causes of its occurrence and development [21]. Therefore, finding new molecules for early diagnosis of HCC, and developing new and effective targeted drugs are problems that need to be solved urgently.

More and more studies have confirmed that abnormally expressed lncRNA plays a dual role (oncogenes or tumor suppressor) in the progression of various tumors [22]. Increasing evidence suggested that some lncRNAs were abnormally expressed during the occurrence of HCC, and their dysregulation correlated to the clinicopathological characteristics of HCC patients [23]. ZEB1-AS1, which originally was discovered in human hepatoma cells, is located on chromosome 10p11.22 and is an antisense transcript of the ZEB1 promoter region [15]. Later, ZEB1-AS1 was found to act as an oncogene in multiple tumors, including prostate cancer, colorectal cancer, bladder cancer, osteosarcoma, glioma, esophageal squamous cell carcinoma, and B lymphocytic leukemia [14]. Li et al. reported that ZEB1-AS1 was obviously upregulated in 102 HCC specimens (especially in metastatic HCC tissue) and HCC cell lines, and the ZEB1-AS1 overexpression promoted cell proliferation and invasion by increasing ZEB1 expression [15]. Moreover, Ma et al. reported that ZEB1-AS1 could promote HCC bone metastasis by inhibiting miR-302b to enhance EGFR-PI3K-AKT signaling [24]. In this study, it was also observed that ZEB1-AS1 expression was abnormally elevated in HCC, and the cell proliferation and invasion were significantly reduced by the inhibition of ZEB1-AS1. We attempt to identify novel mechanisms on the function of ZEB1-AS1 in HCC.

Some studies found that lncRNA could act as ceRNA by completely binding to miRNA or promoting protein stability at the post-transcription level to regulate the occurrence and development of cancer [25]. This study confirmed that miR-23c is a target of ZEB1-AS1 through bioinformatics analysis, and the expression of ZEB1-AS1 and miR-23c in HCC tissues was negatively correlated. MiR-23c was proposed as a tumor suppressor in HCC. Zhang et al. found miR-23c overexpression hampered the tumor growth of HCC by suppressing ERBB2IP expression [26]. They also confirmed KTN1-AS1 facilitated the tumor growth of HCC via miR-23c/ERBB2IP axis [27]. Moreover, Li et al. reported that SNHG5 acted as a ceRNA of miR-23c to modulate HMGB2 expression in HCC [28]. Here, we observed that inhibition of ZEB1-AS1 promoted the miR-23c expression in HCC cells. In addition, the rescue experiments revealed that miR-23c inhibitor transfection counteracted the suppressing effect of ZEB1-AS1 inhibition on the HCC cells proliferation and invasion. Hence, we considered that ZEB1-AS1 affected cell proliferation and invasion by targeting miR-23c in HCC.

Therefore, we confirmed that ZEB1-AS1 had a specific high expression in HCC and regulated proliferation and invasion activities of HCC cells through targeting miR-23c. Collectively, we discovered the important role of ZEB1-AS1 in HCC development, but how ZEB1-AS1 regulates miR-23c and downstream target genes are yet to be further studied. However, our study had several limitations. The sample size is small, and the sample size can be expanded in the future. The effect of ZEB1-AS1 overexpression on HCC cells has not been introduced. In vivo animal experiments have not been performed to verify the phenotypic function of ZEB1-AS1 knockdown. These deficiencies will be supplemented by the follow-up research.

## Conclusion

In summary, we demonstrated that ZEB1-AS1 expression was highly expressed, while miR-23c expression was decreased in human HCC tissues and cells. It was confirmed that miR-23c is a direct target gene of ZEB1-AS1 in HCC cell lines. Therefore, ZEB1-AS1 acted as an oncogene in HCC and negatively regulated miR-23c expression to affect the proliferation and invasion of HCC. These conclusions not only enrich the molecular mechanism of HCC, but also provide new ideas for the prevention and molecular treatment of HCC in the future.

## Data Availability

All data generated or analyzed during this study are included in this published article.
